# The Multiplication of Vaccines

**Published:** 1895-12-28

**Authors:** 


### The Multiplication of Vaccines.

Anti-choleraic vaccination may or may not be a proved success; into this question we do not propose just now to enter. What we wish to do is to draw attention to the prospect opened up to our view by the reports as to the success of his method made by Dr. Haffkine in his recent lecture.

It must be remembered that vaccination, as the term is used, does not mean the introduction of chemical antidotes into the system, but is the implantation within the healthy body of a living virus. Even granting, then, that such an implantation is successful in warding off disease, we have to face the very important question, how far such a manner of protection is desirable as a general principle, and whether this is the most hopeful direction in which bacteriology can aid humanity.

A century ago small-pox was a horrible reality, as, in fact, it still is when once it gets a hold on any population, and for protection from it we thankfully accept vaccination. Nevertheless, it is impossible not to see that the continued acceptance of vaccination as a necessary although irksome part of the process of baby-rearing, is due to its being an isolated fact. By empirical experience we are taught that it will prevent small-pox, and its use has so far led to nothing further. Once, however, enunciate the general principle in regard to other infectious disorders, and the matter will stand on a very different footing; and so it is that, while we do not wish to hold back from the general chorus of praise with which Dr. Haffkine's successes, if they are successes, in vaccinating against cholera have been hailed, we still think it right to raise the much larger question whether this is likely to prove a really useful line of research, or one on which preventive medicine should be encouraged to proceed.

If it were true that every person had to go through every form of infectious disease, and that by vaccination it was possible to give just the required amount of immunity to prevent this consummation, the matter would be somewhat different. But it is notorious that a large number of people entirely escape, and when we have to consider not the individual but the race, remembering all the time how important is that attribute which goes by the rough and ill-defined name "vitality," and how greatly a nation's future depends on each individual, however perfect his veneer of civilisation, having within his skin the making of a strong and healthy savage, we cannot but look with hesitation on the prospect of any extension into a system of what we have put up with, even with thankfulness, so long as it was an isolated affair, namely, the tampering with healthy bodies by their inoculation with morbid germs.

Science is restless; unexpected advances are being made in every quarter; and any day we may awake to find ourselves confronted with a feasible offer of immunity from scarlet fever or diphtheria by means of vaccination. In this there would be great plausibility, for a modicum of immunity which would protect during childhood would practically secure safety, and we cannot doubt that many anxious parents would flock to those who offered safety to their little ones on such terms, especially if backed by the President of the Royal College of Physicians.

If, however, we consider for a moment what would now have been the condition of England if the warnings given by cholera from sixty to thirty years ago, and by typhoid every year, had been met by anti-choleraic or anti-typhoid vaccinations instead of by the sanitary measures which have tended so greatly to the general health of the people, we see at once how disastrously to the community such a means of protection might prove, for, by making the individual independent of the evils around him, the greatest possible incentive to sanitary righteousness would be removed. Preventive vaccination has always been an interesting problem, but we venture to suggest that it is not in that direction, but rather in that of treatment, that bacteriology must look for its future triumphs. Prevention must in the end fall to the sanitarian, the provider of healthy and pure surroundings, the builder of a strong and vital race; the bacteriologist, on the other hand, will find his work in isolating definite chemical compounds by which to render harmless such diseases as may break through the defences set up by the social and sanitary reformer.

